# Draft Genome Sequence of *Rhodococcus triatomae* Strain BKS 15-14

**DOI:** 10.1128/genomeA.00129-13

**Published:** 2013-03-28

**Authors:** Shailesh Kumar, Monu Bala, Gajendra Pal Singh Raghava, Shanmugam Mayilraj

**Affiliations:** a Microbial Type Culture Collection and Gene Bank (MTCC), Chandigarh, India; b Bioinformatics Centre, CSIR-Institute of Microbial Technology, Chandigarh, India

## Abstract

We report the 5.8-Mb genome sequence of *Rhodococcus triatomae* BKS 15-14, isolated from an ant hill soil sample, collected from Bhitarkanika Mangrove Reserve Forest, Odisha, India. The draft genome of strain BKS 15-14 consists of 5,824,349 bp, with a G+C content of 69%, 5,387 protein-coding genes, and 57 RNAs.

## GENOME ANNOUNCEMENT

Members of the *Rhodococcus* genus are well known for their significant ability to degrade numerous xenobiotic compounds. This genus was first proposed by Zopf (1891) and emended by Tsukamura in 1974 (1) and by Goodfellow and Alderson in 1977 (2). *Rhodococcus triatomae* was first reported by A. F. Yassin in 2005, isolated from a blood-sucking bug of the genus *Triatoma* (3). *R. triatomae* is a Gram-positive, aerobic, and coccoid-shaped bacterium. Strain BKS 15-14 is capable of producing cholesterol oxidase, which degrades cholesterol into 4-cholesten-3-one.

The genome of *R. triatomae* BKS 15-14 was sequenced using the Illumina-HiSeq 1000 paired-end technology and produced a total of 29,789,490 paired-end reads (insert size of 350 bp) of 101 bp. We have used the next-generation sequencing quality-control (NGS QC) toolkit v2.3 (4) to filter the data for high-quality (HQ) (cutoff read length for HQ = 70%, cutoff quality score = 20) vector- and adaptor-free reads for genome assembly. A total of 28,013,328 high-quality vector-filtered reads (~565.8× coverage) were used for assembly with Velvet 1.2.08 (at a hash length of 55) (5). The final assembly contains 74 contigs of total size 5,824,349 bp, with an N_50_ contig length of 182 kb; the largest contigs assembled measure 480.6 kb.

The draft genome (74 contigs) comprising 5,824,349 nucleotides (nt) was annotated with the help of the Rapid Annotations using Subsystems Technology (RAST) (6) and RNAmmer 1.2 (7) servers. A total of 5,387 predicted coding regions (CDSs), 2 rRNAs, and 55 tRNAs were predicted. RAST annotation shows that strains *Rhodococcus jostii* RHA1 (score 528), *Rhodococcus opacus* B4 (score 472), and *Rhodococcus erythropolis* PR4 (score 433) are the closest neighbors of BKS 15-14. RAST annotation also shows that strain BKS 15-14 contains the genes coding for glycolysis and gluconeogenesis, the tricarboxylic acid (TCA) cycle, the pentose phosphate pathway, trehalose biosynthesis, a fatty acid metabolic cluster, and glycerolipid and glycerophospholipid metabolism in bacteria. Strain BKS 15-14 has genes coding for butyryl-coenzyme A (CoA) dehydrogenase (EC 1.3.99.2), 3-ketoacyl-CoA thiolase (EC 2.3.1.16), aldehyde dehydrogenase (EC 1.2.1.3), acetyl-CoA acetyltransferase (EC 2.3.1.9), 3-oxoacyl-(acyl-carrier-protein) reductase (EC 1.1.1.100), long-chain-fatty-acid—CoA ligase (EC 6.2.1.3), and enoyl-CoA hydratase (EC 4.2.1.17). In the RAST annotation, we have also found the genes coding for a cholesterol oxidase precursor (EC 1.1.3.6) (CHOD) and cholesterol oxidase (EC 1.1.3.6).

### Nucleotide sequence accession numbers.

The draft genome of *R. triatomae* BKS 15-14 has been included in the GenBank Whole-Genome Shotgun (WGS) database under the accession no. AODO00000000. The version described in this paper is the first version, accession no. AODO01000000.
